# Sexual and gender identity differences in mental health concerns as a function of social media use and cyberbullying among U.S. youth

**DOI:** 10.1111/jora.70295

**Published:** 2026-10-17

**Authors:** Yaxin Pu, Jessica N. Fish

**Affiliations:** ^1^ Department of Human Development and Family Studies University of Illinois Urbana‐Champaign Illinois USA

**Keywords:** adolescents, cyberbullying, gender identity, sexual orientation, social media use

## Abstract

As adolescents increasingly engage with social media, concerns have emerged about its potential implications for youth mental health and about disparities in digital experiences by sexual orientation (SO) and gender identity (GI). However, the mechanisms linking social media use (SMU) to mental health and SOGI disparities in digital contexts remain unclear. Using nationally representative data from the 2023 Youth Risk Behavior Survey (YRBS; *n* = 12,773), the current study examines associations between SMU, cyberbullying, and mental health concerns (i.e., sadness and suicidal ideation), with attention to SO and GI differences in these processes. Results showed that associations between higher SMU and greater risks of sadness and suicidal ideation were largely explained by exposure to cyberbullying. SO and GI disparities were also evident in digital risks and mental health concerns: Despite similar levels of SMU frequency, sexual and gender minority youth reported greater exposure to cyberbullying and greater mental health concerns compared to their heterosexual and cisgender peers, respectively. In addition, sexual and gender minority youth experienced more pronounced mental health impacts when cyberbullying occurred, indicating a potential manifestation of minority stress processes, whereby bias‐based harassment may be particularly harmful to youth mental health concerns. These findings highlight the importance of interventions that move beyond regulating social media use to instead address harassment and cyberbullying in order to promote safer online environments for youth, particularly sexual and gender minority youth.

## INTRODUCTION

In recent years, social media—online communication networks that enable users to create their own content and engage with large and small audiences, synchronously or asynchronously (Bayer et al., 2020)—has become deeply embedded in adolescents' everyday lives. Recent Pew Research Center data indicate that internet and social media use are nearly ubiquitous among U.S. teens: 96% report using the internet daily, and 46% report being online almost constantly (Pew Research Center, 2024). U.S. teens also engage with a wide range of social media platforms, including TikTok, Instagram, and Snapchat; three‐quarters use YouTube at least once a day, and approximately 20% report almost constant social media use (Pew Research Center, 2025). This widespread use is developmentally meaningful, as adolescence is marked by active identity exploration, heightened sensitivity to peer relationships and social evaluation, and an increasing reliance on social contexts for self‐definition and sense of belonging (Steinberg & Morris, 2001). As these developmental tasks increasingly extend into digital environments, social media offers unique opportunities for identity construction and social connection and has become an important context in which adolescents negotiate identity and peer relationships (Ehrenreich et al., 2021; Swirsky et al., 2021). These developmental functions may be particularly salient for sexual and gender minority youth, who engage with social media more frequently than cisgender heterosexual peers (GLSEN, CiPHR, CCRC, 2013; Nagata et al., 2023, 2024), and often rely on online spaces for sexual orientation (SO) and gender identity (GI) exploration and community connection (Craig et al., 2021).

Importantly, adolescence is a sensitive developmental period during which mental health challenges become increasingly prevalent (Hua et al., 2024; McGorry et al., 2025). In the U.S., concerns about youth mental health, especially increased rates of depression and suicidality, have become so pronounced that leading pediatric organizations have declared a national emergency in child and adolescent mental health (American Academy of Pediatrics, American Academy of Child and Adolescent Psychiatry, Children's Hospital Association, 2021). Nationally representative surveys estimate that 40% of high school students in the U.S. report persistent feelings of sadness or hopelessness, and 20% report suicidal ideation, with sexual and gender minority youth reporting more than twice the odds of cisgender heterosexual peers (Centers for Disease Control and Prevention, 2024a). Since social media now serves as a key developmental context for adolescents, it is important to understand how social media use (SMU) relates to mental health concerns and whether these associations differ across SO and GI groups (Office of the Surgeon General, 2021). Such evidence can inform theory, practice, and policy aimed at supporting adolescent well‐being in digital contexts.

### Social media use and adolescent mental health

Prior research examining the associations between the frequency of SMU and adolescent mental health has produced mixed findings. Some studies report positive associations between greater SMU and adolescent well‐being and mental health (e.g., Apaolaza et al., 2013; Swirsky et al., 2021), whereas others find inverse associations (e.g., Barthorpe et al., 2020; Cayubit et al., 2023) or null or mixed effects (e.g., Jelenchick et al., 2013; Thorisdottir et al., 2019). These inconsistencies suggest that the mental health implications of SMU depend less on how frequently adolescents use social media and more on how they engage with it and the characteristics of their online experiences.

On the one hand, social media can provide developmental benefits for young people. SMU may facilitate youth's social connection, identity exploration and expression, and access to information and support (Best et al., 2014; Pretorius et al., 2019). These benefits may be especially salient for sexual and gender minority youth, who often rely on online spaces to explore sexual orientation and gender identity, find community, and access affirming resources that may be unavailable offline (Craig et al., 2015, 2021; Fish et al., 2022).

On the other hand, online environments may also expose youth to developmental risks, including bullying, which is commonly defined as the repeated physical, psychological, or emotional harm to another person, often including an ongoing pattern where power is wielded (Olweus, 1993). Cyberbullying is a distinct experience within youth's broader victimization ecology, but also shares key features with face‐to‐face bullying, including intentionality, repetition, and a high chance of occurring alongside offline bullying (Jose et al., 2012; Lazuras et al., 2017; Waasdorp & Bradshaw, 2015). At the same time, cyberbullying can take distinct forms, such as flaming (e.g., online insults), hacking another person's account, posting denigrating messages, or sending threatening texts (Giumetti & Kowalski, 2022), and may be amplified by features of digital environments, including anonymity, disinhibition, a broader audience, and adolescents' lower likelihood of reporting online harassment to adults (Ehrenreich et al., 2021; Waasdorp & Bradshaw, 2015).

As youth increasingly connect with peers online, opportunities for cyberbullying exposure may increase. Consistent with this possibility, a large‐scale study of 180,919 adolescents across 42 countries suggests that more frequent SMU was positively associated with cyberbullying (Craig et al., 2020). In the United States, approximately 10% of early adolescents (ages 10–14) report experiencing cyberbullying, with roughly two‐thirds reporting an occurrence in the past 12 months (Nagata et al., 2022). Consistent with in‐person experiences of bullying, cyberbullying is consistently associated with poorer adolescent well‐being and mental health, including lower life satisfaction, elevated loneliness, depressive and anxiety symptoms, and greater suicidality (e.g., Espinoza et al., 2020; Eyuboglu et al., 2021; Hinduja & Patchin, 2019; Li et al., 2024; Messias et al., 2014; Viner et al., 2019). These concerns are further heightened in the current digital landscape, where changes in platform governance and content moderation increase the potential for online harassment and harmful content, particularly for marginalized groups (Munzert et al., 2025).

Despite these growing concerns, much of the existing literature has focused on how frequently adolescents use social media, with comparatively less attention to the contexts and experiences that occur during that time online. However, the implications of SMU for adolescent mental health may depend less on how often adolescents use social media and more on the nature of their online experiences (Office of the Surgeon General, 2021). Accordingly, examining cyberbullying as a potential pathway linking SMU to adolescent mental health concerns is warranted.

### SOGI differences in digital experiences and risks

Although cyberbullying is a risk for any youth with access to digital technology, exposure to such risk is not evenly distributed. Sexual and gender minority youth consistently report disproportionately high rates of cyberbullying compared with their cisgender heterosexual peers (UNESCO, 2023; Zych et al., 2015). Emerging evidence also indicates that sexual and gender minority youth may engage more frequently with social media than cisgender and heterosexual peers (Nagata et al., 2023, 2024), although this work has largely relied on non‐probability samples. Greater engagement with online environments may increase exposure to potential digital risks for sexual and gender minority youth, which is especially important because online spaces often serve as crucial contexts where they explore their identities and form community connections, and cyberbullying can disrupt or complicate these developmental processes (Mariotto et al., 2026). Analyses from large‐scale U.S. national samples revealed that sexual minority youth have nearly three times greater odds of experiencing cyberbullying than their heterosexual peers, and transgender and gender diverse adolescents face more than twice the risk relative to their cisgender peers (Nagata et al., 2022, 2025). Consequently, sexual and gender minority youth who experience cyberbullying report elevated mental health concerns, including depressive symptoms and suicidal ideation (e.g., Gordon et al., 2026; Liu et al., 2025; Noon et al., 2025; Wright et al., 2022), and some evidence suggests that negative online experiences, including cyberbullying, may exert more pronounced adverse mental health consequences among sexual and gender minority youth than for cisgender heterosexual youth, potentially because chronic experiences of stigma and rejection may heighten their sensitivity to peer rejection and amplify its psychological impact (Bitran et al., 2026; Jones et al., 2026).

Minority stress theory (Meyer, 2003) provides a framework for understanding these patterns, which posits that stigma‐related stressors, such as bullying and discrimination, place sexual and gender minority populations at heightened risk for health disparities. For instance, school‐based bullying has long been identified as a salient stressor for sexual and gender minority youth, with consistently higher prevalence rates documented across countries (e.g., Chan & Lam, 2023; Kaufman et al., 2020; Myers et al., 2020; Sun et al., 2023; Toomey & Russell, 2016). As adolescents' social interactions increasingly extend into digital contexts, these vulnerabilities may likewise manifest online. From this perspective, disparities in negative digital experiences, including cyberbullying, may reflect broader contextual vulnerabilities linked to their marginalized sexual orientation and/or gender identity. At the same time, the distinctive features of cyberbullying, such as anonymity and a broader audience, may amplify harm and warrant focused examination as a distinct digital risk context (Waasdorp & Bradshaw, 2015).

### Current study

Despite emerging evidence documenting SOGI disparities in digital risks and mental health outcomes, nationally representative evidence on these patterns remains limited. In addition, although emerging research has begun to examine the mechanisms through which engagement with digital environments may contribute to SOGI disparities in mental health (e.g., Escobar‐Viera et al., 2020; Jones et al., 2026), there is room to expand understanding in meaningful ways. Addressing these gaps is critical to inform regulatory standards and intervention efforts that strengthen online safety and foster more supportive digital environments for youth. In this vein, we used a nationally representative sample of U.S. adolescents to examine the relationship among SMU, digital experiences, and mental health concerns with explicit attention to differences by SO and GI. We first test whether greater SMU frequency is associated with higher levels of sadness and suicidal ideation (Model a), and the degree to which these associations differ by SO and GI groups (Model b). To further characterize these associations, we examine whether cyberbullying statistically accounted for part of the associations between SMU frequency and mental health concerns (Model c).

Informed by prior theoretical and empirical work, we also evaluate three non‐mutually exclusive rationales that may underlie SOGI disparities in digital risks and mental health concerns. Specifically, we assessed whether (1) sexual and gender minority youth engage in higher levels of SMU overall, potentially increasing exposure to negative digital experiences, including cyberbullying (assessed through group differences in SMU); (2) at comparable levels of SMU, sexual and gender minority youth are more likely than their heterosexual and cisgender peers to experience cyberbullying, which is associated with greater mental health concerns (Model d, *a* path); and (3) cyberbullying is more strongly associated with adverse mental health concerns for sexual and gender minority youth than for heterosexual and cisgender youth (Model d, *b* path). The full conceptual framework is presented in Figure 1.

**FIGURE 1:**
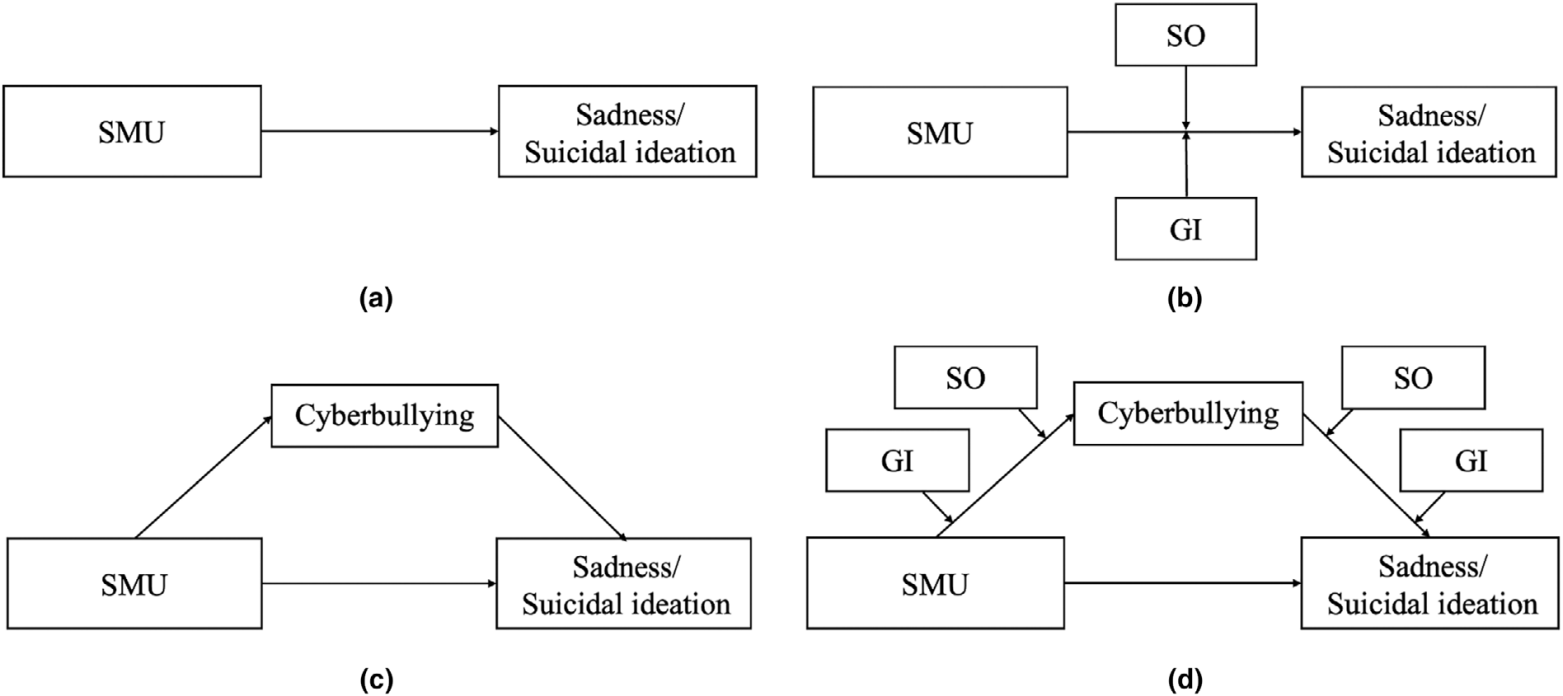
Conceptual model. (a) Main‐effect model; (b) moderation of the SMU‐health association by SO or GI ; (c) indirect effect model via cyberbullying; (d) moderation of the *a* and *b* paths by SO or GI. Sadness and suicidal ideation were estimated as outcome variables in separate models. Sexual orientation and gender identity were examined in separate models to facilitate interpretation of subgroup‐specific effects and reduce model complexity. In Model (d), the moderation of *a* path and *b* path was tested in separate models to isolate pathway‐specific effects. Covariates were included in all models.

## METHOD

### Procedure and participants

This study used secondary data from the 2023 National Youth Risk Behavior Survey (YRBS), conducted biannually by the Centers for Disease Control and Prevention (CDC) in collaboration with state and local education agencies. The survey collects health‐risk behavior data on students in 9th to 12th grades in the U.S. and its territories. The YRBS employs a three‐stage cluster sampling design to produce nationally representative estimates (Centers for Disease Control and Prevention, 2024b). The present secondary analysis of publicly available, de‐identified data was deemed exempt from human subjects review by the University of Illinois Urbana‐Champaign Institutional Review Board.

The 2023 YRBS data source included 20,103 high school students. For this study, participants with missing data on variables of interest were excluded, resulting in a final analytic sample of 12,773 U.S. adolescents.^1^ Imputing missing values for the primary exposure, SMU, would require modeling the unobserved distribution of social media use and preserving its associations with SOGI variables, cyberbullying, mental health concerns, and complex survey design features (Sterne et al., 2009; White et al., 2011). This was nontrivial given the use of ordinal and binary variables, survey‐weighted categorical path models, and interaction terms (Bartlett et al., 2015). We therefore retained the complete‐case analytic subpopulation in survey‐weighted models, allowing the full sample to contribute to estimation of the complex survey design when estimating model parameters for the analytic subpopulation. Among the analytic sample, most participants were aged 15–17 years (76.5%), and 49.9% were female. Regarding sexual orientation, 76.7% identified as heterosexual, 3.5% as gay or lesbian, 15.1% as bisexual or other sexual identity, and 4.6% as questioning. The majority identified as cisgender (95.1%), with 2.8% identifying as transgender and 2.1% as questioning. The sample was racially and ethnically diverse (45.2% White, 9.7% Black, 22.9% Hispanic/Latino, 5.1% Asian, and 17.1% multiracial or other). Social media use was common: 80.2% reported using it several times per day or more; 5.8% of youth reported no use. Weighted demographics are presented in Supplemental Materials, Table S1.

### Measures

#### Sexual orientation and gender identity

Sexual orientation was assessed with the item, “Which of the following best describes you?” Response options included *Heterosexual (straight)*, *Gay or lesbian*, *Bisexual*, *I describe my sexual identity some other way* (coded as other identity), *I am not sure about my sexual identity (questioning)* (coded as questioning), and *I do not know what this question is asking* (coded as missing value). Adolescents identifying as bisexual and those reporting another sexual identity showed similar scores across key study variables, so they were combined into a single category (“Bisexual/other”) for the analysis to retain power (Table S2).

Gender identity was measured by the question: “Some people describe themselves as transgender when their sex at birth does not match the way they think or feel about their gender. Are you transgender?” Participants indicated their response as: *No, I am not transgender* (coded as cisgender); *Yes, I am transgender* (coded as transgender); *I am not sure if I am transgender* (coded as questioning); or *I do not know what this question is asking* (coded as missing value). Transgender and questioning respondents showed similar scores across key study variables (Table S3), so these categories were combined for analysis to retain power.

#### Social media use

SMU was measured by a question asking, “How often do you use social media?” Response options included: *I do not use social media*, *A few times a month*, *About once a week*, *A few times a week*, *About once a day*, *Several times a day*, *About once an hour*, and *More than once an hour*. For analysis, responses were recoded into an ordinal, approximately continuous scale reflecting increasing frequency of use: 1 = never, 2 = less than once a day, 3 = once a day, 4 = several times a day, 5 = once an hour, 6 = more than once an hour.

#### Cyberbullying

Cyberbullying was measured by a question: “During the past 12 months, have you ever been electronically bullied? (Includes being bullied through texting, Instagram, Facebook, or other social media.)” Responses were dichotomous (yes = 1, no = 0) and retained as binary indicators for analysis.

#### Sadness and suicidal ideation

Participants were asked the question pertaining to their experiences in the past 12 months: (1) “Did you ever feel so sad or hopeless almost every day for two weeks or more in a row that you stopped doing some usual activities?” (sadness); and (2) “Did you ever seriously consider attempting suicide?” (suicidal ideation). Responses were dichotomous (yes = 1, no = 0) and retained as binary indicators for analysis.

#### Covariates

Demographic covariates included age (coded as an ordinal variable ranging from 14 years or younger [reference] to 18 years or older), sex (male [reference] and female), and race/ethnicity (categorized as White [reference], Black, Hispanic/Latino, Asian, Multiple, and Other [American Indian/Alaska Native and Native Hawaiian/Other Pacific Islander]). School‐based bullying (“During the past 12 months, have you ever been bullied on school property?” coded as yes [1] or no [0]) was also modeled as a covariate given its established associations with higher levels of adverse mental health outcomes, particularly for sexual and gender minority youth (Chan & Lam, 2023; Myers et al., 2020; Sun et al., 2023; Toomey & Russell, 2016).

### Analytic strategy

All analyses used survey‐weighted procedures to account for the YRBS's complex sampling design, including clustering, stratification, and unequal selection probabilities. Sampling weights, primary sampling units (PSUs), and strata were specified using R and Mplus survey commands, with adjustments for single‐unit strata to ensure valid variance estimation. Statistical significance was evaluated at *p* < .05.

Descriptive analyses first estimated weighted population distributions of key study variables and differences across SO and GI groups. Weighted means and proportions were calculated for SMU, cyberbullying, sadness, and suicidal ideation. Group differences were evaluated using design‐adjusted Wald tests for continuous outcomes and Rao‐Scott‐corrected *F* tests for categorical outcomes. To examine associations between SMU and mental health concerns, survey‐weighted regression models were estimated separately for sadness and suicidal ideation, with SMU as the primary predictor, adjusting for age, sex, race/ethnicity, and school‐based bullying. Moderation analyses tested whether these associations differed by SO and GI by including interaction terms between SMU and SO (heterosexual [reference], lesbian/gay, bisexual/other, questioning) and between SMU and GI (cisgender [reference], transgender/questioning). Indirect pathways in the relation between SMU and mental health concerns through cyberbullying were examined by estimating associations between SMU and cyberbullying, cyberbullying and mental health concerns, and the remaining direct associations of SMU with outcomes, controlling for covariates. Indirect effects were estimated using a weighted least‐squares estimator, with the delta method used to obtain standard errors and CIs appropriate for binary variables (Muthén & Muthén, 2012). Effect modification by SO and GI was tested in separate models.

Sensitivity analyses were conducted to assess the robustness of findings to potential conceptual overlap across key constructs. First, all regression and indirect effect models were re‐estimated without adjustment for school‐based bullying to assess for potential confounding (Waasdorp & Bradshaw, 2015). Second, to address potential overlap between SO and GI, models examining SO differences additionally adjusted for GI, and models examining GI differences additionally adjusted for SO. These analyses evaluated whether observed effects remained consistent when both identity dimensions were considered simultaneously. We also examined sex differences and found that although several constructs differed by sex, interaction tests did not show that the main associations varied by sex; therefore, primary analyses were performed on the full sample with sex included as a covariate (Tables S4 and S5).

## RESULTS

### Descriptive statistics

Our sample showed widespread social media use, with 79% reporting use at least several times a day (see Supplemental Materials, Table S1). SMU did not significantly differ by SO or GI groups, except that lesbian/gay youth use social media slightly less often than heterosexual peers (*M*_lesbian/gay_ = 3.99, 95% CI [3.82, 4.17]; *M*_heterosexual_ = 4.28, 95% CI [4.22, 4.34]; see Table 1). However, results revealed SO and GI differences in negative digital experiences and mental health concerns: sexual and gender minority youth reported significantly higher prevalence in cyberbullying, sadness, and suicidal ideation than heterosexual and cisgender youth, respectively (all *p*s < .001; see Table 1).

**TABLE 1:** Differences in key variables across SO and GI subgroups.

	SMU, *M* [95% CI]	Cyberbullying, % [95% CI]	Sadness, % [95% CI]	Suicidal ideation, % [95% CI]
Sexual orientation	*F* = 4.49**	*F* = 30.74***	*F* = 169.38***	*F* = 172.19***
Heterosexual	4.28 [4.22, 4.34]	13.2 [12.1, 14.3]	31.8 [29.9, 33.7]	13.2 [12.0, 14.4]
Lesbian/gay	3.99 [3.82, 4.17]	23.5 [18.4, 29.4]	61.9 [54.7, 68.6]	33.2 [26.7, 40.5]
Bisexual/other	4.34 [4.24, 4.44]	26.0 [22.0, 30.3]	68.0 [64.9, 70.9]	41.2 [38.7, 44.9]
Questioning	4.37 [4.15, 4.60]	19.1 [14.7, 24.4]	57.0 [50.2, 63.6]	32.7 [27.2, 38.8]
Gender Identity	*F* = 1.32	*F* = 65.78***	*F* = 102.82***	*F* = 184.67***
Cisgender	4.29 [4.24, 4.35]	14.9 [13.7, 16.3]	37.9 [35.9, 39.9]	17.5 [16.2, 18.9]
Transgender/questioning	4.14 [3.86, 4.42]	32.4 [27.6, 37.6]	72.6 [66.2, 78.3]	50.9 [44.8, 56.9]

*Note*: Values are survey‐weighted means or percentages with 95% confidence intervals. Group differences were evaluated using design‐adjusted Wald tests for continuous outcomes and Rao–Scott corrected *F* tests for categorical outcomes, which adjust Pearson's *χ* ^2^ for the complex survey design. **p < .01, ***p < .001.

Abbreviation: SMU, social media use.

### Associations between SMU and mental health concerns and moderation of SO/GI

Regression models indicated that greater SMU was associated with higher levels of both sadness (*B* = .08, SE = .01, *p* < .001) and suicidal ideation (*B* = .03, SE = .02, *p* = .034) among youth, supporting our expectation. School‐based bullying was also strongly and positively associated with both outcomes (*B* = .80, SE = .05, *p* < .001, and *B* = .76, SE = .05, *p* < .001, respectively). However, interactions between SMU and SO were not statistically significant for sadness (*B*_SMU*lebian/gay_ = .01, SE = .06, *p* = .896; *B*_SMU*bisexual/other_ = −.00, SE = .04, *p* = .906; *B*_SMU*questioning_ = .03, SE = .06, *p* = .633) or suicidal ideation (*B*_SMU*lesbian/gay_ = .00, SE = .07, *p* = .991; *B*_SMU*bisexual/other_ = .00, SE = .04, *p* = .987; *B*_SMU*questioning_ = −.04, SE = .05, *p* = .405); interactions between SMU and GI were also not statistically significant for either outcome (*B* = .03, SE = .05, *p* = .543; *B* = .02, SE = .05, *p* = .676, respectively), suggesting that the association between SMU and mental health concerns did not differ by SO or GI.

### Indirect associations through cyberbullying

Indirect effect models (see Table 2), estimated separately for sadness and suicidal ideation, revealed that greater SMU was significantly associated with a higher likelihood of cyberbullying (*B* = .10, SE = .02, *p* < .001). Cyberbullying, in turn, was associated with higher levels of both sadness (*B* = .35, SE = .03, *p* < .001) and suicidal ideation (*B* = .30, SE = .03, *p* < .001).

**TABLE 2:** Indirect effect models.

Path	*B* (SE)	*p*	95% CI
Dependent variable: sadness			
SMU → cyberbullying	.10 (.02)	< .001	[.07, .13]
Cyberbullying → sadness	.35 (.03)	< .001	[.28, .40]
SMU → sadness (direct)	.04 (.01)	< .001	[.02, .05]
SMU → cyberbullying → sadness (indirect)	.04 (.01)	< .001	[.02, .06]
Dependent variable: suicidal ideation			
SMU → cyberbullying	.10 (.02)	< .001	[.07, .13]
Cyberbullying → suicidal ideation	.30 (.03)	< .001	[.25, .35]
SMU → suicidal ideation (direct)	.00 (.02)	.851	[−.03, .03]
SMU → cyberbullying → suicidal ideation (indirect)	.03 (.01)	< .001	[.02, .04]

*Note*: Age, sex, race, and school‐based bullying were controlled. Coefficients are unstandardized. Indirect effects were estimated using the WLSMV delta method.

Significant indirect associations were observed for both outcomes, supporting our hypothesis. That is, SMU was indirectly associated with both greater sadness (*B* = .04, 95% CI [.02, .06], *p* < .001) and suicidal ideation (*B* = .03, 95% CI [.02, .04], *p* < .001) via cyberbullying. The direct association between SMU and sadness remained significant (*B* = .04, SE = .01, *p* < .001), whereas the direct association between SMU and suicidal ideation was not statistically significant (*B* = .00, SE = .02, *p* = .851) in the presence of the indirect effect.

### Conditional effects by SO/GI

Conditional models indicated that the SMU → cyberbullying (*a*) path differed by GI groups (*B* = .13, *p* = .048), such that transgender and gender diverse adolescents experience a stronger association between social media use and cyberbullying than their cisgender peers. However, this association did not vary statistically across SO groups (*B*s = −.00 to .13, *p*s = .051 to .956), which was inconsistent with our hypothesis that sexual minority youth experience greater exposure to cyberbullying at comparable levels of SMU.

Findings from the moderation analysis on the cyberbullying → mental health concerns (*b*) path showed evidence of subgroup differences. Regarding SO, the association between cyberbullying and sadness was significantly stronger among lesbian/gay (*B* = .61, SE = .22, *p* = .007) and bisexual/other youth (*B* = .62, SE = .11, *p* < .001) than heterosexual youth (Figure 2a), whereas the interaction for questioning youth did not differ (*B* = .43, SE = .28, *p* = .127). Similarly, compared to heterosexual youth, cyberbullying was more strongly associated with suicidal ideation among bisexual/other youth (*B* = .34, SE = .09, *p* < .001; see Figure 2b), but not among lesbian/gay (*B* = .31, SE = .20, *p* = .130) or questioning youth (*B* = .11, SE = .23, *p* = .623). The interaction between cyberbullying and gender identity was not significant for sadness (*B* = .17, SE = .23, *p* = .462), but was significant for suicidal ideation (*B* = .29, SE = .15, *p* = .046; Figure 2c), indicating a stronger association between cyberbullying and suicidal ideation among transgender and gender diverse adolescents compared to cisgender youth. Altogether, these findings provide partial support for the hypothesis that sexual and gender minority youth exhibit greater mental health vulnerability related to cyberbullying.

**FIGURE 2:**
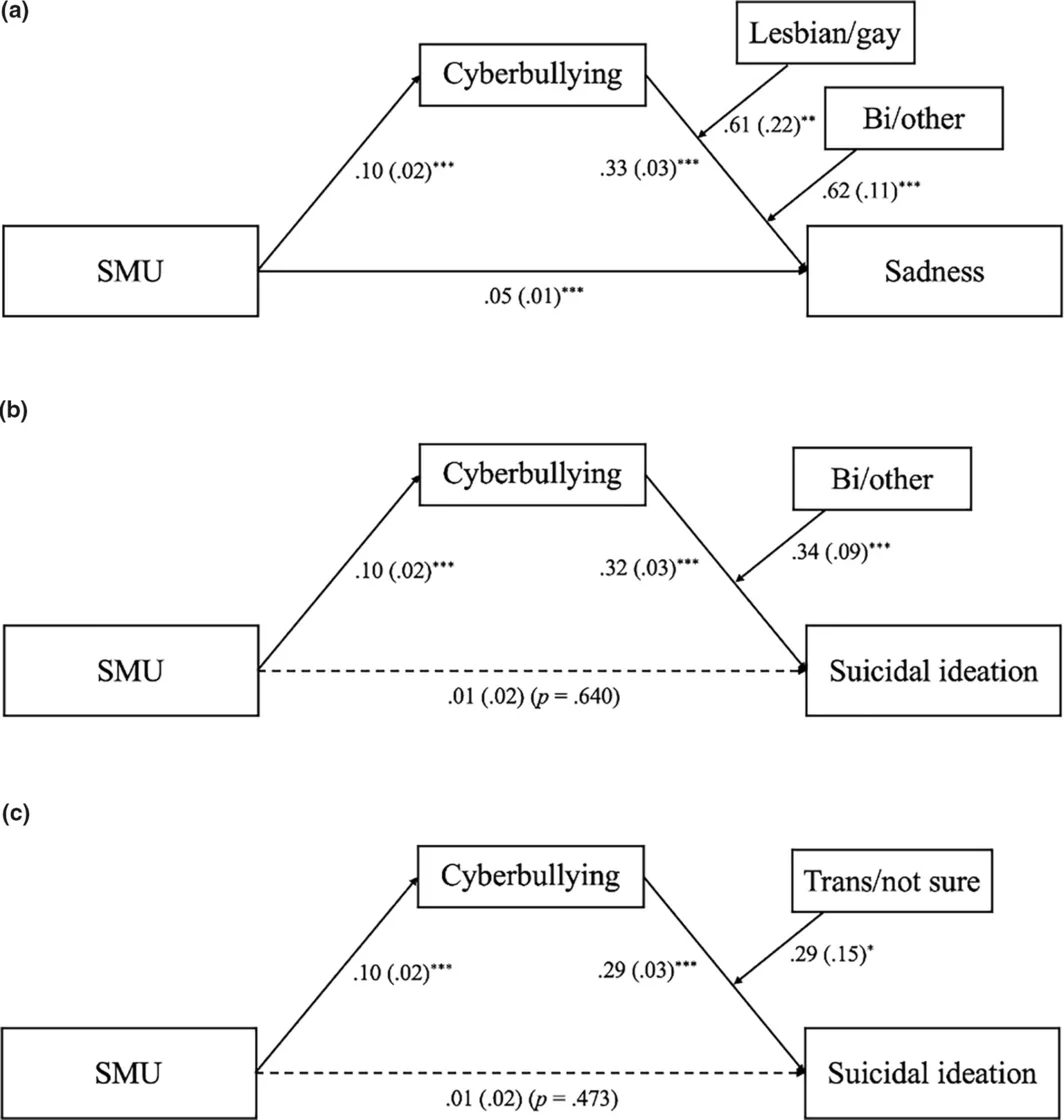
(a–c). Conditional indirect pathways linking SMU to mental health concerns across SO and GI groups. Panel a shows pathways to sadness (moderated by SO); Panel b shows pathways to suicidal ideation (moderated by SO); Panel c shows pathways to suicidal ideation (moderated by GI). Unstandardized survey‐weighted coefficients are shown. Covariates were included in all models; only significant moderation effects are displayed. ^*^ *p* < .05, ^**^ *p* < .01, ^***^ *p* < .001.

### Sensitivity analyses

Sensitivity analyses that exclude school‐based bullying from models a, b, and c (see Figure 1) yielded substantively similar results (Tables S6–S9). Notably, after removing school bullying, the associations between cyberbullying and both sadness and suicidal ideation became stronger, suggesting both unique and shared variance across youths' experiences of cyberbullying and school‐based bullying. Additional sensitivity analyses accounting for the potential overlap between SO and GI yielded similar results to the primary models (Tables S10–S12). The only exception was the moderating role of GI on SMU → cyberbullying (*a*) path, which was statistically significant in the primary model but became non‐significant after controlling for SO.

## DISCUSSION

Our study aimed to examine the associations among SMU frequency, cyberbullying experiences, and mental health concerns, with particular attention to SO and GI disparities in these processes. Using a nationally representative U.S. sample, we found that more frequent SMU was associated with higher levels of sadness and suicidal ideation among adolescents, and that these associations operated indirectly through greater exposure to cyberbullying. Consistent with previous research, we observed that sexual and gender minority youth displayed greater exposure to cyberbullying and greater mental health concerns than their heterosexual and cisgender peers (Fish et al., 2023; Russell & Fish, 2016; UNESCO, 2023; Zych et al., 2015), although the association between SMU frequency and cyberbullying did not differ across SO or GI. Importantly, sexual and gender minority youth appeared more vulnerable to the adverse mental health consequences of cyberbullying when it occurred.

Our findings are consistent with other national data that social media use is highly prevalent among U.S. adolescents: in the 2023 YRBS, more than three‐quarters of youth in the analytic sample reported using social media several times per day or more. Recent data from the Pew Research Center indicate that 96% of U.S. teens report using the internet daily and 46% report being online almost constantly (Pew Research Center, 2024). These patterns underscore that frequent online engagement is a routine part of adolescents' developmental ecology, where youth navigate core developmental tasks such as building social connections, seeking information, and exploring identity (Ehrenreich et al., 2021; Steinberg & Morris, 2001; Swirsky et al., 2021).

Given the mixed findings in the extant literature regarding associations between SMU frequency and youth mental health (e.g., Apaolaza et al., 2013; Barthorpe et al., 2020; Cayubit et al., 2023; Jelenchick et al., 2013; Swirsky et al., 2021; Thorisdottir et al., 2019), this study moved beyond examining the direct association between SMU and mental health concerns by testing cyberbullying as a potential, modifiable pathway through which SMU confers mental health risk. Our findings revealed that more frequent SMU was linked with greater sadness and suicidal ideation primarily through greater risks of cyberbullying. These results align with existing literature suggesting that greater engagement with social media can increase exposure to cyberbullying (Craig et al., 2020), a well‐established risk factor for adolescent well‐being and mental health (e.g., Espinoza et al., 2020; Eyuboglu et al., 2021; Hinduja & Patchin, 2019; Li et al., 2024; Viner et al., 2019). Taken together, these findings support the view that the mental health implications of SMU might depend less on the frequency of use and more on the quality of adolescents' online experiences, suggesting that public concerns should shift from an oversimplified approach to reducing youth SMU frequency to preventing harmful online environments.

Our findings also revealed clear SO and GI disparities in negative digital experiences and mental health concerns. Sexual and gender minority youth reported greater exposure to cyberbullying as well as higher levels of sadness and suicidal ideation compared with their heterosexual and cisgender peers. These findings are consistent with prior research documenting elevated interpersonal risks and mental health vulnerabilities among sexual and gender minority youth (e.g., Fish et al., 2023; Russell & Fish, 2016). As adolescents increasingly extend their social lives into digital environments, online spaces appear to mirror and reproduce existing social inequalities. By documenting these disparities in social media contexts, our findings provide empirical support for minority stress theory within digital environments (Meyer, 2003). Notably, our analyses indicated that cyberbullying represents a distinct form of risk, above and beyond the effects of school‐based bullying. Despite their frequent co‐occurrence and shared mental health correlates (Jose et al., 2012; Lazuras et al., 2017; Waasdorp & Bradshaw, 2015), features unique to cyberbullying, such as anonymity and a broader audience, may create distinct harm in digital contexts (Waasdorp & Bradshaw, 2015), again underscoring the need for strategies to reduce online bullying and other harmful content (e.g., hate speech).

We further examined potential SO and GI differences in these processes. Contrary to prior findings based on non‐representative samples (Nagata et al., 2023, 2024), not all sexual and gender minority youth subgroups in our study reported higher levels of social media use than their cisgender and heterosexual peers (e.g., gay and lesbian youth), indicating that the disparities in digital risks are unlikely to stem solely from differences in SMU frequency. In addition, unexpectedly, across primary and sensitivity analyses, models showed inconsistent evidence suggesting that increases in SMU were associated with comparable levels of cyberbullying across SO and GI groups. One potential explanation is that SMU frequency does not capture qualitative differences in how youth engage with digital environments. Sexual and gender minority youth may be more likely to seek out platforms or communities that provide sexual and gender identity affirmation and social connection (Craig et al., 2015, 2021; Fish et al., 2022), where they do not necessarily experience higher risks of cyberbullying at comparable levels of SMU. Therefore, although identity‐related stigma may place sexual and gender minority youth at a higher overall risk of cyberbullying, each increase in SMU may not translate into a correspondingly larger increase in victimization. A second explanation concerns the study's measurement and data distribution: SMU was assessed using broad ordinal frequency categories, with most adolescents concentrated in the highest‐use categories, whereas cyberbullying was measured as a dichotomous past‐year experience. This restricted variability and difference in measurement precision may have reduced the analyses' sensitivity to detect relatively small subgroup differences in the SMU–cyberbullying association.

Although we did not observe SO or GI differences in exposure to cyberbullying at comparable levels of SMU, we did find notable differences in the association between cyberbullying and sadness and suicidal ideation: Compared to their heterosexual and cisgender peers, lesbian/gay, bisexual/other, and transgender/questioning youth, respectively, appear more susceptible to the emotional toll of cyberbullying, aligning with findings from prior research (Bitran et al., 2026). That is, the association between cyberbullying and sadness and suicidal ideation was stronger for sexual and gender minority youth relative to their cisgender and heterosexual peers. Together, these findings suggest that disparities in digital contexts may arise less from differences in social media exposure and more from differences in vulnerability to harmful online experiences (e.g., cyberbullying). One possible explanation is that cyberbullying may function as a digital manifestation of minority stress processes, in which identity‐specific harassment (i.e., bias‐based bullying) carries heightened psychological impact for marginalized youth, particularly sexual and gender minority youth (Russell et al., 2012). In other words, sexual and gender minority youth are likely to experience bias‐based bullying on social media related to their minoritized identity, which likely has more deleterious impacts on mental health relative to more general forms of cyberbullying.

Of note, despite the stronger mental health consequences of cyberbullying among sexual and gender minority youth, the overall association between SMU and mental health concerns was not stronger for them than for heterosexual and cisgender youth. One possible explanation is that social media may also provide developmental benefits that partially offset negative experiences. For example, many sexual and gender minority youth use social media to explore and develop their LGBTQ+ identity, seek emotional and social support, build a sense of community belonging, and access affirming resources that may be unavailable offline (Craig et al., 2015, 2021; Fish et al., 2022). These positive experiences may complement or counterbalance some of the adverse effects of negative online interactions (Bitran et al., 2026), helping explain why greater SMU was not uniquely detrimental for sexual and gender minority youth overall.

## IMPLICATIONS

These findings have several implications for efforts to promote adolescent well‐being in digital environments. First, the results suggest that policy and prevention efforts should move beyond generalized concerns about how much time youth spend on social media. In this study, associations between social media use and mental health concerns were largely explained by adolescents' exposure to cyberbullying, indicating that the risks of social media may depend more on harmful online experiences than on the amount of time spent online. Accordingly, efforts to promote youth well‐being should prioritize preventing peer bullying and harassment in digital contexts and strengthening protections against cyberbullying and other harmful content. Given the distinct features of cyberbullying, school‐based bullying prevention programs may need to extend their focus to online environments. In addition, schools and families can play an important role in promoting digital literacy by equipping youth with strategies to manage harmful online experiences, such as blocking or reporting users, curating content, and adjusting privacy settings, which may help reduce exposure to cyberbullying and mitigate its impact.

In addition, these findings highlight the importance of addressing disparities in online safety for sexual and gender minority youth. Although this group did not consistently report higher levels of social media use or greater exposure to cyberbullying at comparable levels of SMU than their cisgender and heterosexual peers, they appeared to experience more cyberbullying overall and greater mental health impacts when cyberbullying occurred. This pattern suggests that disparities in mental health concerns may arise not only from unequal exposure to risks but also from unequal vulnerability to the harms associated with those risks. From this perspective, cyberbullying may be more discriminatory (e.g., identity‐ or bias‐based), functioning as a form of minority stress in digital contexts. Prevention efforts should therefore incorporate strategies that explicitly address identity‐based harassment, including hate speech and other discriminatory content, and support the safety and inclusion of diverse youth in online environments. Expanding inclusive education in schools, strengthening digital anti‐bullying policies, improving content moderation standards, and promoting affirming online communities may all contribute to reducing disparities in youth mental health concerns.

## LIMITATIONS AND FUTURE DIRECTIONS

Despite our contribution, there are important limitations to consider. First, the YRBS data are cross‐sectional, which limits our ability to draw causal conclusions about the relationships among social media use, cyberbullying, and mental health concerns. Although the analyses provide insight into potential pathways linking these factors, longitudinal research is needed to understand the temporal ordering of social media use, online bullying, and mental health concerns among adolescents. Second, measures of bullying and mental health concerns (sadness and suicidal ideation) were assessed using single‐item indicators. These measures limit our ability to adequately capture the frequency and severity of adolescents' experiences. Considering the complexity of cyberbullying, future research should involve more detailed assessments that identify different types and frequency of cyberbullying, including firsthand experiences, indirect exposure to harmful online content, witnessing bullying of others, and identity‐specific online harm. This approach could help clarify how various online experiences and related factors influence adolescent mental health. Third, social media use was measured solely by frequency and did not capture the type, context, or content of adolescents' online engagement. Although we examined cyberbullying as one concrete and consequential negative experience within the social media context, the YRBS social media use item does not distinguish between different patterns of digital activity or identify which specific online experiences shape cyberbullying exposure and mental health concerns. This limitation may be particularly important for sexual and gender minority youth, who may seek out identity‐affirming communities and supportive content even as they remain vulnerable to stigma and harassment in other digital spaces (Craig et al., 2015, 2021; Fish et al., 2022). Future research should incorporate more detailed assessments of digital behaviors and online experiences to better understand the mechanisms of how different forms of engagement contribute to youth mental health concerns across SOGI groups. Fourth, SMU had a high degree of missing data (24.4%), although the rationale for this high degree of missing data on this item is not documented in the YRBS data files. Unfortunately, it may be that these data are not missing completely at random and therefore could introduce some degree of error in our model. We hope that others will be able to replicate our findings to support our claims. Lastly, future research should also move beyond covariate adjustment to examine how other sociodemographic characteristics, such as race/ethnicity and age, may shape sexual minority adolescents' digital experiences and mental health in meaningful ways.

## CONCLUSION

Using nationally representative data from U.S. adolescents, this study examined associations between SMU frequency, cyberbullying, and mental health, with a particular focus on SO and GI differences in these processes. Results indicated that the relationship between SMU and mental health concerns was largely explained by adolescents' exposure to cyberbullying, suggesting specific concerns beyond social media use frequency itself. In addition, although sexual and gender minority youth did not consistently report higher levels of SMU or greater exposure to cyberbullying at comparable levels of SMU, they experienced greater prevalence of cyberbullying and more pronounced mental health impacts from exposure to cyberbullying. These findings suggest that disparities in digital contexts may arise not only from differences in exposure to risk but also from differences in vulnerability to its consequences. As digital environments continue to shape adolescents' social lives, efforts to promote youth's well‐being should focus on reducing cyberbullying and fostering safer, more inclusive online spaces for all youth.

## AUTHOR CONTRIBUTIONS

**Yaxin Pu:** Conceptualization; methodology; investigation; formal analysis; visualization; writing – original draft; writing – review and editing. **Jessica N. Fish:** Conceptualization; methodology; supervision; writing – review and editing.

## FUNDING INFORMATION

The authors have nothing to report.

## CONFLICT OF INTEREST STATEMENT

The authors declare that they have no conflicts of interest with respect to their authorship or the publication of this article.

## ETHICS STATEMENT

This study used publicly available, de‐identified secondary data from the 2023 Youth Risk Behavior Survey (YRBS), which was approved by the CDC Institutional Review Board and is exempt from further IRB review. Therefore, the ethics approval statement is not applicable.

## CONSENT

Patient consent is not applicable for this study.

## Supporting information


**Table S1**. Analytic sample characteristics, missingness, and comparison with excluded respondents.
**Table S2**. Key variables by original sexual orientation categories.
**Table S3**. Key variables by original gender identity categories.
**Table S4**. Key variables by sex.
**Table S5**. Moderation by sex.
**Table S6**. Main effects regression models without controlling for school‐based bullying.
**Table S7**. Moderation of SO without controlling for school‐based bullying.
**Table S8**. Moderation of GI without controlling for school‐based bullying.
**Table S9**. Indirect effect models without controlling for school‐based bullying.
**Table S10**. Moderation of SO on SMU → Mental health, controlling GI.
**Table S11**. Moderation of GI on SMU → Mental health, controlling SO.
**Table S12**. Moderated pathways in indirect effect model (model d), controlling for SO and GI.

## Data Availability

The data that support the findings of this study are publicly available from the Centers for Disease Control and Prevention (CDC) Youth Risk Behavior Surveillance System (YRBS) at https://www.cdc.gov/healthyyouth/data/yrbs/index.htm.

## Footnotes

^1^
Missingness was concentrated in SMU (*n* = 4900, 24.4% of the full sample). Preliminary diagnostic checks indicated no systematic clustering of missingness in key study variables within sampling units, providing no evidence that item‐level nonresponse was driven by survey administration patterns. Respondents included in and excluded from the analytic subpopulation were similar in sex, race/ethnicity, school‐based bullying, cyberbullying, and sadness, but differed in age, sexual orientation, gender identity, SMU, and suicidal ideation. Full survey‐weighted distributions and design‐adjusted comparisons are presented in Supplemental Materials, Table S1. Since the excluded subsample had higher weighted proportions of sexual and gender minority youth than the analytic subsample, the analytic subsample may have reduced SGMY representation.
