# Supplementary material for: Sexual and gender identity differences in mental health concerns as a function of social media use and cyberbullying among U.S. youth
Source: J Res Adolesc. 2026 Oct 17;36(4):e70295. doi: 10.1111/jora.70295 (PMC13675716; doi:10.1111/jora.70295)
Supplement: Supplementary file 1 [file JORA-36-0-s001.docx]

**Sexual and Gender Identity Differences in Mental Health Concerns as a Function of Social Media Use and Cyberbullying among U.S. Youth**

**Supplemental Materials**

**Table S1** Analytic sample characteristics, missingness, and comparison with excluded respondents

| Variable | Sample (*n*, %) | Sample (%_w_) | Excluded (%_w_) | *p* |
| --- | --- | --- | --- | --- |
| **Age** | Missingness: 98 (0.5) |  |  |  |
| 14 or younger | 1,493 (11.7) | 11.4 | 15.6 | .022 |
| 15 | 3,492 (27.3) | 25.6 | 24.4 |  |
| 16 | 3,258 (25.5) | 25.5 | 27.2 |  |
| 17 | 3,021 (23.7) | 25.1 | 22.7 |  |
| 18 or older | 1,509 (11.8) | 12.4 | 10.1 |  |
| **Sex** | Missingness: 158 (0.8) |  |  |  |
| Male | 6,393 (50.1) | 51.7 | 52.2 | .806 |
| Female | 6,380 (49.9) | 48.3 | 47.8 |  |
| **Race/ethnicity** | Missingness: 370 (1.8) |  |  |  |
| White | 5,775 (45.2) | 47.5 | 49.8 | .592 |
| Black | 1,233 (9.7) | 12.4 | 15.6 |  |
| Hispanic/Latino | 2,926 (22.9) | 28.8 | 24.0 |  |
| Asian | 652 (5.1) | 4.1 | 4.7 |  |
| Multiracial | 1,270 (9.9) | 6.5 | 5.2 |  |
| Other | 917 (7.2) | 0.7 | 0.8 |  |
| **Sexual orientation** | Missingness: 2,468 (12.3) |  |  |  |
| Heterosexual | 9,800 (76.7) | 76.6 | 70.6 | .007 |
| Lesbian/Gay | 451(3.5) | 3.7 | 5.7 |  |
| Bisexual/Other | 1,930 (15.1) | 15.5 | 18.1 |  |
| Questioning | 592 (4.6) | 4.2 | 5.6 |  |
| **Gender identity** | Missingness: 2,077 (10.3) |  |  |  |
| Cisgender | 12,146 (95.1) | 95.1 | 92.4 | < .001 |
| Transgender/Questioning | 627 (4.9) | 4.9 | 7.6 |  |
| **Social media use** | Missingness: 4,900 (24.4) |  |  |  |
| Never | 747 (5.8) | 6.0 | 20.3 | < .001 |
| Less than once a day | 1,008 (7.9) | 7.9 | 21.3 |  |
| Once a day | 770 (6.0) | 6.2 | 5.0 |  |
| Several times a day | 5,124 (40.1) | 41.8 | 25.9 |  |
| Once an hour | 999 (7.8) | 7.8 | 4.3 |  |
| More than once an hour | 4,125 (32.3) | 30.3 | 23.1 |  |
| **School bullying** | Missingness: 201 (1.0) |  |  |  |
| Yes | 2,560 (20.0) | 18.8 | 20.2 | .655 |
| No | 10,213 (80.0) | 81.2 | 79.8 |  |
| **Cyberbullying** | Missingness: 205 (1.0) |  |  |  |
| Yes | 2,173 (17.0) | 15.8 | 17.4 | .620 |
| No | 10,600 (83.0) | 84.2 | 82.6 |  |
| **Sadness** | Missingness: 240 (1.2) |  |  |  |
| Yes | 5,285 (41.4) | 39.6 | 40.1 | .823 |
| No | 7,488 (58.6) | 60.4 | 59.9 |  |
| **Suicidal ideation** | Missingness: 436 (2.2) |  |  |  |
| Yes | 2,710 (21.2) | 19.2 | 23.8 | .048 |
| No | 10,063 (78.8) | 80.8 | 76.2 |  |

*Note.* The full sample included 20,103 respondents; the complete-case analytic sample included 12,773 respondents, and 7,330 respondents were excluded. Missingness is shown for the full sample. Percentages (%) are unweighted, %_w_ are survey-weighted. *P*-values are from design-adjusted Rao-Scott *F* tests comparing included versus excluded respondents, using available non-missing data for each variable.

**Table S2.** Key variables by original sexual orientation categories.

|  | %_w_ |  |  |  |  |  |
| --- | --- | --- | --- | --- | --- | --- |
| Variable | Straight | Gay/lesbian | Bisexual | Other | Questioning | *F* |
| Social media use, *M* | 4.28 | 3.99 | 4.40 | 4.17 | 4.37 | 4.49^**^ |
| Cyberbullying |  |  |  |  |  |  |
| Yes | 13.2 | 23.5 | 25.3 | 27.7 | 19.1 | 20.65^***^ |
| No | 86.8 | 76.5 | 74.7 | 72.3 | 80.9 |  |
| School-based bullying |  |  |  |  |  |  |
| Yes | 15.7 | 34.7 | 29.6 | 30.4 | 21.8 | 19.61^***^ |
| No | 84.3 | 65.3 | 70.4 | 69.6 | 78.2 |  |
| Sadness |  |  |  |  |  |  |
| Yes | 31.8 | 61.9 | 66.5 | 72.3 | 57.0 | 113.09^***^ |
| No | 68.2 | 38.1 | 33.5 | 27.7 | 43.0 |  |
| Suicidal ideation |  |  |  |  |  |  |
| Yes | 13.2 | 33.2 | 41.4 | 42.8 | 32.7 | 121.05^***^ |
| No | 86.8 | 66.8 | 58.6 | 57.2 | 67.3 |  |

*Note.* ^*^*p* < .05, ^**^*p* < .01, ^***^*p* < .001

**Table S3.** Key variables by original gender identity categories.

|  | %_w_ |  |  |  |
| --- | --- | --- | --- | --- |
| Variable | Cisgender | Transgender | Questioning | *F* |
| Social media use, *M* | 4.29 | 4.18 | 4.08 | 0.75 (*p* = .478) |
| Cyberbullying |  |  |  |  |
| Yes | 14.9 | 33.5 | 30.8 | 34.19^***^ |
| No | 85.1 | 66.5 | 69.2 |  |
| School-based bullying |  |  |  |  |
| Yes | 17.8 | 39.5 | 40.0 | 31.66^***^ |
| No | 82.2 | 60.5 | 60.0 |  |
| Sadness |  |  |  |  |
| Yes | 37.9 | 72.1 | 73.4 | 65.66^***^ |
| No | 62.1 | 27.9 | 26.6 |  |
| Suicidal ideation |  |  |  |  |
| Yes | 17.5 | 55.4 | 44.7 | 103.33^***^ |
| No | 82.5 | 44.6 | 55.3 |  |

*Note.* ^*^*p* < .05, ^**^*p* < .01, ^***^*p* < .001

**Table S4.** Key variables by sex.

| Variable | Female (%_w_) | Male (%_w_) | *F* |
| --- | --- | --- | --- |
| Social media use, *M* | 4.44 | 4.14 | 61.35^***^ |
| Cyberbullying |  |  | 97.23^***^ |
| Yes | 20.3 | 11.6 |  |
| No | 79.7 | 88.4 |  |
| School-based bullying |  |  | 38.91^***^ |
| Yes | 21.7 | 16.2 |  |
| No | 78.3 | 83.8 |  |
| Sadness |  |  | 282.71^***^ |
| Yes | 52.8 | 27.2 |  |
| No | 47.2 | 72.8 |  |
| Suicidal ideation |  |  | 112.68^***^ |
| Yes | 25.5 | 13.2 |  |
| No | 74.5 | 86.8 |  |

*Note.* ^*^*p* < .05, ^**^*p* < .01, ^***^*p* < .001

**Table S5.** Moderation by sex.

| Path | *B* (*SE*) | *p* |
| --- | --- | --- |
| SMU × Sex → Cyberbullying | .08 (.06) | .199 |
| SMU × Sex → Sadness | .02 (.04) | .697 |
| SMU × Sex → Suicidal ideation | -.01 (.05) | .920 |
| SMU × Sex → Sadness (adjusted for cyberbullying) | .01 (.04) | .846 |
| SMU × Sex → Suicidal ideation (adjusted for cyberbullying) | -.02 (.05) | .736 |
| Cyberbullying × Sex → Sadness | -.23 (.12) | .061 |
| Cyberbullying × Sex → Suicidal ideation | -.15 (.18) | .396 |

*Note.* Each row reports the interaction term testing whether sex moderated the specified association. Male was coded as 0 and female as 1. Models were survey-weighted and adjusted for age, race/ethnicity, school-based bullying, sexual orientation, and gender identity. ^*^*p* < .05, ^**^*p* < .01, ^***^*p* < .001

**Table S6.** Main effects regression models without controlling school-based bullying.

|  | Dependent Variable | | | |
| --- | --- | --- | --- | --- |
|  | Sadness | | Suicidal ideation | |
| Variable | *B* (*SE*) | *p* | *B* (*SE*) | *p* |
| SMU | .09 (.01) | < .001 | .05 (.02) | .002 |
| Age | .01 (.02) | .763 | .02 (.02) | .450 |
| Sex (ref = male) | .66 (.04) | < .001 | .45 (.04) | < .001 |
| Race (ref = White) |  |  |  |  |
| Black | -.01 (.04) | .803 | -.11 (.06) | .070 |
| Latino | .03 (.05) | .538 | -.18 (.05) | .001 |
| Asian | -.32 (.08) | < .001 | -.36 (.08) | < .001 |
| Multiple | .02 (.09) | .799 | -.01 (.08) | .885 |
| Other | -.19 (.17) | .269 | -.26 (.23) | .252 |

*Note.* Coefficients are unstandardized.

**Table S7.** Moderation of SO without controlling school-based bullying.

|  | Dependent Variable | | | |
| --- | --- | --- | --- | --- |
|  | Sadness | | Suicidal ideation | |
| Variable | *B* (*SE*) | *p* | *B* (*SE*) | *p* |
| SMU | .10 (.01) | < .001 | .06 (.02) | .003 |
| Sexual orientation |  |  |  |  |
| Lesbian/gay | .77 (.09) | < .001 | .65 (.10) | < .001 |
| Bisexual/other | .80 (.04) | < .001 | .82 (.04) | < .001 |
| Questioning | .50 (.09) | < .001 | .60 (.08) | < .001 |
| SMU * Lesbian/gay | .01 (.07) | .907 | .00 (.07) | .999 |
| SMU * Bisexual/other | -.00 (.04) | .968 | .01 (.04) | .860 |
| SMU * Questioning | .02 (.06) | .788 | -.05 (.05) | .268 |
| Age | .00 (.02) | .988 | .01 (.02) | .656 |
| Sex (ref = male) | .51 (.04) | < .001 | .27 (.04) | < .001 |
| Race (ref = White) |  |  |  |  |
| Black | -.02 (.04) | .617 | -.13 (.06) | .045 |
| Latino | .06 (.05) | .175 | -.16 (.05) | .002 |
| Asian | -.28 (.08) | < .001 | -.34 (.08) | < .001 |
| Multiple | .02 (.09) | .864 | -.04 (.08) | .643 |
| Other | -.14 (.17) | .413 | -.22 (.24) | .349 |

*Note.* Coefficients are unstandardized.

**Table S8.** Moderation of GI without controlling school-based bullying.

|  | Dependent Variable | | | |
| --- | --- | --- | --- | --- |
|  | Sadness | | Suicidal ideation | |
| Variable | *B* (*SE*) | *p* | *B* (*SE*) | *p* |
| SMU | .09 (.01) | < .001 | .05 (.02) | < .001 |
| Transgender/questioning | .87 (.10) | < .001 | .90 (.08) | < .001 |
| SMU * Transgender/questioning | .03 (.05) | .482 | .02 (.04) | .587 |
| Age | .00 (.02) | .809 | .01 (.02) | .508 |
| Sex (ref = male) | .63 (.04) | < .001 | .43 (.04) | < .001 |
| Race (ref = White) |  |  |  |  |
| Black | .02 (.04) | .632 | -.07 (.06) | .233 |
| Latino | .04 (.05) | .354 | -.16 (.05) | .001 |
| Asian | -.30 (.08) | < .001 | -.34 (.08) | < .001 |
| Multiple | .03 (.09) | .785 | -.01 (.08) | .905 |
| Other | -.16 (.17) | .346 | -.22 (.22) | .321 |

*Note.* Coefficients are unstandardized.

**Table S9.** Indirect effect models without controlling school-based bullying.

| Path | *B* (*SE*) | *p* | 95% CI |
| --- | --- | --- | --- |
| Dependent variable: Sadness |  |  |  |
| SMU → Cyberbullying | .03 (.01) | .001 | [.01, .04] |
| Cyberbullying → Sadness | .87 (.05) | < .001 | [.77, .98] |
| SMU → Sadness (direct) | .06 (.01) | < .001 | [.04, .09] |
| SMU → Cyberbullying → Sadness (indirect) | .02 (.01) | .002 | [.01, .04] |
| Dependent variable: Suicidal ideation |  |  |  |
| SMU → Cyberbullying | .03 (.01) | .001 | [.01, .04] |
| Cyberbullying → Suicidal ideation | .77 (.04) | < .001 | [.69, .85] |
| SMU → Suicidal ideation (direct) | .03 (.02) | .077 | [-.00, .06] |
| SMU → Cyberbullying →Suicidal ideation (indirect) | .02 (.01) | .001 | [.01, .03] |

*Note.* Age, sex, and race were controlled. Coefficients are unstandardized. Indirect effects were estimated using the WLSMV delta method.

**Table S10.** Moderation of SO on SMU → Mental health, controlling GI.

|  | Dependent Variable | | | |
| --- | --- | --- | --- | --- |
|  | Sadness | | Suicidal ideation | |
| Variable | *B* (*SE*) | *p* | *B* (*SE*) | *p* |
| SMU | .10 (.01) | < .001 | .06 (.02) | .002 |
| Sexual orientation |  |  |  |  |
| Lesbian/gay | .67 (.09) | < .001 | .52 (.10) | < .001 |
| Bisexual/other | .73 (.04) | < .001 | .73 (.04) | < .001 |
| Questioning | .45 (.09) | < .001 | .54 (.08) | < .001 |
| SMU * Lesbian/gay | .01 (.07) | .914 | -.00 (.07) | .965 |
| SMU * Bisexual/other | .00 (.04) | .913 | .01 (.04) | .733 |
| SMU * Questioning | .01 (.06) | .905 | -.07 (.05) | .174 |
| Transgender/questioning | .40 (.09) | < .001 | .48 (.08) | < .001 |
| Age | .00 (.02) | 1.000 | .01 (.02) | .657 |
| Sex (ref = male) | .51 (.04) | < .001 | .27 (.04) | < .001 |
| Race (ref = White) |  |  |  |  |
| Black | -.01 (.04) | .867 | -.10 (.06) | .103 |
| Latino | .06 (.05) | .159 | -.15 (.05) | .002 |
| Asian | -.28 (.08) | < .001 | -.33 (.08) | < .001 |
| Multiple | .02 (.09) | .851 | -.03 (.08) | .670 |
| Other | -.14 (.17) | .426 | -.20 (.23) | .390 |

*Note.* Coefficients are unstandardized.

**Table S11.** Moderation of GI on SMU → Mental health, controlling SO.

|  | Dependent Variable | | | |
| --- | --- | --- | --- | --- |
|  | Sadness | | Suicidal ideation | |
| Variable | *B* (*SE*) | *p* | *B* (*SE*) | *p* |
| SMU | .10 (.01) | < .001 | .06 (.02) | .001 |
| Transgender/questioning | .40 (.10) | < .001 | .48 (.08) | < .001 |
| SMU * Transgender/questioning | .01 (.05) | .792 | .00 (.05) | .969 |
| Sexual orientation |  |  |  |  |
| Lesbian/gay | .67 (.09) | < .001 | .52 (.10) | < .001 |
| Bisexual/other | .73 (.04) | < .001 | .73 (.04) | < .001 |
| Questioning | .45 (.08) | < .001 | .54 (.07) | < .001 |
| Age | .00 (.02) | .999 | .01 (.02) | .638 |
| Sex (ref = male) | .51 (.04) | < .001 | .27 (.04) | < .001 |
| Race (ref = White) |  |  |  |  |
| Black | -.01 (.04) | .879 | -.10 (.06) | .096 |
| Latino | .07 (.05) | .156 | -.15 (.05) | .002 |
| Asian | -.28 (.08) | < .001 | -.33 (.08) | < .001 |
| Multiple | .02 (.09) | .849 | -.03 (.08) | .687 |
| Other | -.13 (.17) | .453 | -.20 (.23) | .397 |

*Note.* Coefficients are unstandardized.

**Table S12.** Moderated pathways in indirect effect model (model d), controlling for SO and GI.

| Path | Adjusted *B*(*SE*) | *p* |
| --- | --- | --- |
| SMU * Lesbian/gay → Cyberbullying | -.01 (.06) | .873 |
| SMU * Bisexual/other → Cyberbullying | .01 (.05) | .885 |
| SMU * Questioning → Cyberbullying | .13 (.13) | .305 |
| SMU * Transgender/questioning → Cyberbullying | .13 (.07) | .056 |
| Cyberbullying * Lesbian/gay → Sadness | .59 (.22) | .006 |
| Cyberbullying * Bisexual/other → Sadness | .61 (.11) | < .001 |
| Cyberbullying * Questioning → Sadness | .39 (.27) | .141 |
| Cyberbullying * Transgender/questioning → Sadness | .22 (.25) | .384 |
| Cyberbullying * Lesbian/gay → Suicidal ideation | .30 (.19) | .122 |
| Cyberbullying * Bisexual/other → Suicidal ideation | .32 (.09) | < .001 |
| Cyberbullying * Questioning → Suicidal ideation | .08 (.23) | .715 |
| Cyberbullying * Transgender/questioning → Suicidal ideation | .31 (.15) | .036 |

*Note.* Models examining SO differences included GI as a covariate, and models examining GI differences included SO as a covariate. Reported coefficients correspond to interaction terms estimating the moderation of the association between SMU and cyberbullying (*a* path) and between cyberbullying and mental health concerns (*b* path). Sadness and suicidal ideation were estimated in separate models. All models adjust for age, sex, race/ethnicity, and school-based bullying. Coefficients are unstandardized.
